# Supplementary material for: Hepatic zonation determines tumorigenic potential of mutant β-catenin
Source: Nature. 2025 Nov 19;649(8097):739–48. doi: 10.1038/s41586-025-09733-1 (PMC12804091; doi:10.1038/s41586-025-09733-1)
Supplement: Supplementary file 1 — Supplementary table includes sequences of the DNA oligo pool used for rRNA depletion in preparing samples for Ribosome Sequencing. [file 41586_2025_9733_MOESM1_ESM.pdf]

nature portfolio

https://doi.org/10.1038/s41586-025-09733-1

Supplementary information

Hepatic zonation determines tumorigenic potential of mutant β-catenin

In the format provided by the
authors and unedited

rRNA_depl_1	GCATGTATTAGCTCTAGAATTACCACAGTTATCCAAGTA
rRNA_depl_2	GAAACCCCGACCCAGAAGCAGGTCGTCTACGAATGG
rRNA_depl_3	GACCCTGCTTAGCTTCCGAGATCAGACGAGATCGGGCGC
rRNA_depl_4	GAGCCATTCGCAGTTTCACTGTA
rRNA_depl_5	CCCTTCACCTTCATTGCGCCACG
rRNA_depl_6	CAAAGGAACCATAACTGATTTAATGAGCCATTCGCAGTT
rRNA_depl_7	GGACCGGCTATCCGAGGCCAACCGAGG
rRNA_depl_8	AGCTGCTCTGCTACGTACGAAACCCCGACCCAGAAGCAGG
rRNA_depl_9	GGATCTGAACCCGACTCCCTTTCGATCGGCCGAGGGCA
rRNA_depl_10	GAGGTTATCTAGAGTCACCAAAGCCG
rRNA_depl_11	AAGCAGGTCGTCTACGAATGGTTTAG
rRNA_depl_12	GGGACCCGGCTATCCGGGGCCAACCGAGG
rRNA_depl_13	ATTATTCCTAGCTGCGGTATCC
rRNA_depl_14	GCCACTTGTCCCTCTAAGAAGT
rRNA_depl_15	TACCGGCCTCACACCGTCCACGGGC
rRNA_depl_16	GCCAGGTTCCACACGAACGTGCGTTC
rRNA_depl_17	CGCTGAGCCAGTCAGTGTAGCGCGCGTGCAGCCCCGGACATCT
rRNA_depl_18	AGCATGCCAGAGTCTCGTTCGTTATCGGAATTAACCAGACAAATCGCTCCACCAACT
rRNA_depl_19b	TTCTTCATCGACGCACGAGCCGAGTGATCCACCGCT
rRNA_depl_20	AATCATTCGCTTTACCGGATAAAACTGCGTACGTCGGGAGCGAGAGCGCCAGCTATCCTGAG
rRNA_depl_21	GGGCTCTTCCCTGTTCACTCGCCGTT
rRNA_depl_23	GCCCATCTCTCAGGACCGACTGACCCAT
rRNA_depl_24	ACAAACCCTTGTGTCGAGGGCTGACTTTCAAT
rRNA_depl_25	ACTGGGCAGAAATCACATCGCGTCAACACCCGCCGCGGGCCTTCGCGAT
rRNA_depl_26	GGGCCTCCCACTTATTCTACACCTCTCATGTCTCTTCACC
rRNA_depl_27	GGTTAGTTTCTTTTCCTCCGCTGACTAATATGCTTAAATTCAGCGGGTCGCCACGTCT
rRNA_depl_28	AAGAGAGTCATAGTTACTCCCGCCGTTT
rRNA_depl_29	ACCCACGGAATCGAGAAAGAGCTATCAATCTGTCAATCCTGTCCGT
rRNA_depl_30	GGACTCATTCCAATTACAGGGCCTC
rRNA_depl_31	GTCTGAACCTGCGGTTCCTCTCGT
rRNA_depl_32	ACGGATCCGGCTTGCCGACTTCCCTTACCTACATTGTTCCAACATGCCAGAGGCT
rRNA_depl_33	GCCCTTCTGCTCCACGGGAGGTTTCTGTCCTCCCTGAGCTCGCCTT
rRNA_depl_34	AACACTCGGGGTGAGGTGGTTCGGCGCG
rRNA_depl_35	GATCTGATAAATGCACGCATCCCCCCCG
rRNA_depl_36	GGTTTCCCGGAAGCTGCCCGGCGGGTCATGG
rRNA_depl_37	ATTGGCTCCTCAGCCAAGCACAT
rRNA_depl_38	ACAGCACCCGGTATTCCCAGGCGGTCTCCCATCCAAGTACT
rRNA_depl_39	GCTGGCACCAGACTTGCCCTCCAAT
rRNA_depl_40	GCTTAAAACCCAAAAGGTCAGAAGGATCGT
rRNA_depl_41	ACCGGGTCAGTGAAAAAACGATGAGAGTAGTGGTATTTCACCGGCGGC
rRNA_depl_42	ATCGGTCTCGTGCCGGTATTTAGCCTT
rRNA_depl_43	AGTTGATTCGGCAGGTGAGTTGTT
rRNA_depl_44	AGCGACGCTCAGACAGGCGTAGCCCCGGGAG
rRNA_depl_45	AGATAGTCAAGTTCGACCGTCTTCTCAGC
rRNA_depl_46	CCGACCCCGGCGGGGCCGATCCGAGGGCCTCACT
rRNA_depl_47	GAGCAGGATTACCATGGCAACAAC
rRNA_depl_48	ACCTCTTAACGGTTTCACGCCCTCTT
rRNA_depl_49	GACCGCCCGCCCGCTCCCAAGATCCAACT
rRNA_depl_50	ACAAAGGGCAGGGACTTAATCAAC
rRNA_depl_51	GACCCGCACTTACTGGGAATTCCTCGTTCAT

rRNA_depl_52	ACGAACGGCTCTCCGCACCGGACCCCGGTCCCG
rRNA_depl_53	GGGAATCTCGTTCATCCATTCAT
rRNA_depl_54	AATTAGATGACGAGGCATTTGGCT
rRNA_depl_55	ACCCGGGGCCGCAAGTGCGTTCGAAGTGTCGAT
rRNA_depl_56	ATTAGTGGGTGAACAATCCAACGCTT
rRNA_depl_57	GGGTACGGCCCGGCGCGAGATTT
rRNA_depl_58	ATCCAGGCGGCTCGGGCCTGCTTTGAACACTCT
rRNA_depl_59	GCCCCAGTCAAACTCCCCACCTGGCACT
rRNA_depl_60	GCCCCAGCCCGACCGACCCAGCCCTT
rRNA_depl_61	GCGTTCAGGGTGGTATGGCCGTAGAC
rRNA_depl_62	ATCTGATCGTCTTCGAACCTCCG
rRNA_depl_63	ACCATCGAAAGTTGATAGGGCAGACGTTC
rRNA_depl_64	GACCCGACGGCGCGACGACGCCCGGGGCGCACTGG
rRNA_depl_65	AATCGGTAGTAGCGACGGGCGGTGTGT
rRNA_depl_66	ACCCAGGTCGGACGACCGATTTGCAC
rRNA_depl_67	GGCCTCGATCAGAAGGACTTGGGCCCCCCACG
rRNA_depl_68	ATCGCCAGTCGGCATCGTTTATGGTCGGAACT
rRNA_depl_69	ATCGTTCCGCCTGGGCGGGATTCTGACTT
rRNA_depl_70	GCCCCAGCCCGACCGACCCAGCCCTTAGAGCCAATCCTTATCCCGAAGTT
rRNA_depl_71	GGTGGTGCGCCCTCGGCGGACTGGAGAGGCCTCGGGATCCCACCTCGGC
rRNA_depl_72	GGAAACTTCGGAGGGAACCAGCTACTAGATGGTTCG
rRNA_depl_73	CGACTGCCGGCGACGGCCGGGTATGGGCCCGACGCTCCAGCGCCATCCAT
rRNA_depl_74	ACCCTCTCCCCCGGATTTTCAAGGGCCAGCGAGAGCTCACCGGACGCCGCCG
rRNA_depl_75	CGCCCCGCCGCCCGCCGACCGCCGCCGCCCG
rRNA_depl_76	TTCCCCACGAACGTGCGGTGCGTGACGGGCGAGGGGGCGGCCG
rRNA_depl_77	GGGGCGCCGAGAGGCAAGGGGCGGGGACGGGCGGT
rRNA_depl_78	TCAATGTGTCCTGCAATTCACATTAATTCTCG
rRNA_depl_79	CCCCCGGGCCCGACGGCGCGACCCGCCCGGGGCGCACTG
rRNA_depl_80	AGCTGGGGCGATCCACGGGAAGGGCCCGGCTCGCGTCCAGAGTCGCCGCCGCCGCCG
