# Supplementary material for: Hepatic zonation determines tumorigenic potential of mutant β-catenin
Source: Nature. 2025 Nov 19;649(8097):739–48. doi: 10.1038/s41586-025-09733-1 (PMC12804091; doi:10.1038/s41586-025-09733-1)
Supplement: Supplementary file 2 — Reporting Summary [file 41586_2025_9733_MOESM2_ESM.pdf]

nature portfolio

Corresponding author(s): Owen Sansom

Last updated by author(s): Oct 1, 2025

Reporting Summary

Nature Portfolio wishes to improve the reproducibility of the work that we publish. This form provides structure for consistency and transparency in reporting. For further information on Nature Portfolio policies, see our Editorial Policies and the Editorial Policy Checklist.

Statistics

For all statistical analyses, confirm that the following items are present in the figure legend, table legend, main text, or Methods section.

n/a	Confirmed	
☐	☒	The exact sample size (*n*) for each experimental group/condition, given as a discrete number and unit of measurement
☐	☒	A statement on whether measurements were taken from distinct samples or whether the same sample was measured repeatedly
☐	☒	The statistical test(s) used AND whether they are one- or two-sided<br>*Only common tests should be described solely by name; describe more complex techniques in the Methods section.*
☐	☒	A description of all covariates tested
☐	☒	A description of any assumptions or corrections, such as tests of normality and adjustment for multiple comparisons
☐	☒	A full description of the statistical parameters including central tendency (e.g. means) or other basic estimates (e.g. regression coefficient) AND variation (e.g. standard deviation) or associated estimates of uncertainty (e.g. confidence intervals)
☐	☒	For null hypothesis testing, the test statistic (e.g. *F*, *t*, *r*) with confidence intervals, effect sizes, degrees of freedom and *P* value noted<br>*Give P values as exact values whenever suitable.*
☒	☐	For Bayesian analysis, information on the choice of priors and Markov chain Monte Carlo settings
☐	☒	For hierarchical and complex designs, identification of the appropriate level for tests and full reporting of outcomes
☐	☒	Estimates of effect sizes (e.g. Cohen's *d*, Pearson's *r*), indicating how they were calculated

Our web collection on statistics for biologists contains articles on many of the points above.

Software and code

Policy information about availability of computer code

Data collection	Olympus cellSens imaging software (version 1.7.1), HALO image analysis software (version 2.0.1145, Indica Labs), Columbus software (version 2.9.1.532), ZEN Black image acquisition software (version 2009), GeoMx NGS pipeline on NanoString's DND platform. Leica Aperio ImageScope software (version 12.4.3.5008)
Data analysis	recount3 package (version 1.6), GenomicDataCommons R package (version 1.12.0), DESeq2 (version 1.36), FastQC algorithm (version 0.11.8), mouse genome build GRCm38.98 using HISAT2 (version 2.1.0.), FeatureCounts (version 1.6.4.), DESeq2 (version 1.22.2), ReactomePA (version 1.36.0.), GSA (version 1.03.1), GSVA (version 1.40.1), R (version 4.3.1), Cutadapt (version 1.18), UMI-tools (version 1.0.1), BBmap (version 38.18), samtools (version 1.9), Bowtie2 (version 2.3.5.1), RSEM (version 1.3.3), EnrichR, GraphPad Prism (version 7.0.4). Fiji/ImageJ software (version 1.53t),

For manuscripts utilizing custom algorithms or software that are central to the research but not yet described in published literature, software must be made available to editors and reviewers. We strongly encourage code deposition in a community repository (e.g. GitHub). See the Nature Portfolio guidelines for submitting code & software for further information.

Data

Policy information about availability of data

All manuscripts must include a data availability statement. This statement should provide the following information, where applicable:

- Accession codes, unique identifiers, or web links for publicly available datasets
- A description of any restrictions on data availability
- For clinical datasets or third party data, please ensure that the statement adheres to our policy

The RNA sequencing and spatial transcriptomic data generated in this study are publicly available through the Gene Expression Omnibus (GEO) with the following accession codes; GSE230644, GSE230110, GSE230137, GSE230144, GSE275864.

Research involving human participants, their data, or biological material

Policy information about studies with human participants or human data. See also policy information about sex, gender (identity/presentation), and sexual orientation and race, ethnicity and racism.

Reporting on sex and gender	Not applicable
Reporting on race, ethnicity, or other socially relevant groupings	Not applicable
Population characteristics	Not applicable
Recruitment	Not applicable
Ethics oversight	Not applicable

Note that full information on the approval of the study protocol must also be provided in the manuscript.

Field-specific reporting

Please select the one below that is the best fit for your research. If you are not sure, read the appropriate sections before making your selection.

☒ Life sciences ☐ Behavioural & social sciences ☐ Ecological, evolutionary & environmental sciences

For a reference copy of the document with all sections, see nature.com/documents/nr-reporting-summary-flat.pdf

Life sciences study design

All studies must disclose on these points even when the disclosure is negative.

Sample size	A priori based on historical data sets were used to ensure the smallest sample size that could give a significant difference was chosen in accordance with the 3Rs
Data exclusions	Regarding animal models that used an inducible Cre-lox genetic system, samples were excluded if there was evidence of a failed injection of inducing agents and evidence of impaired genetic recombination.
Replication	Multiple biological replicates were used to verify reproducibility of experiments. All attempts at replication were successful. All experiments were replicated at least twice.
Randomization	For all histological analysis the samples were randomized. For genetic studies, animals were assigned to groups according to their genotype. Treatment groups were randomly assigned however steps were taken during group assignment to avoid separating males in to singly housed cages. Selection of groups also aimed to maintain an equal sex balance.
Blinding	For all histological analysis the samples researchers were blinded to the genotype or treatment. Investigators were blinded during treatment regimens and at sample collection for timepoint experiments. For aging experiments it was not possible for investigators to be blind to genotype as this factor needed to be known to maintain the welfare of the experimental cohort

Reporting for specific materials, systems and methods

We require information from authors about some types of materials, experimental systems and methods used in many studies. Here, indicate whether each material, system or method listed is relevant to your study. If you are not sure if a list item applies to your research, read the appropriate section before selecting a response.

Materials & experimental systems

n/a	Involved in the study
☐	☒ Antibodies
☒	☐ Eukaryotic cell lines
☒	☐ Palaeontology and archaeology
☐	☒ Animals and other organisms
☒	☐ Clinical data
☒	☐ Dual use research of concern
☒	☐ Plants

Methods

n/a	Involved in the study
☒	☐ ChIP-seq
☒	☐ Flow cytometry
☒	☐ MRI-based neuroimaging

Antibodies

Antibodies used

β-catenin (1:50, 610154, BD Biosciences), glutamine synthetase (1:300, ab73593, Abcam (IF); 1:800; HPA007316, Sigma-Aldrich (IHC)), BrdU (1:400, ab6326, Abcam (IF); 1:250, 347580, BD Biosciences (IHC)), HNF4α (1:300, PP-H1415-00, Perseus Proteomics), E-cadherin (1:300, 610181, BD Biosciences), Ki67 (1:1000, 12202, Cell Signaling Technology), IGFBP2 (1:1000, PA5-81409, Invitrogen), RFP (1:1000,0 600-401-379, Rockland), cleaved caspase 3 (1:500, 9661, Cell Signaling Technology), cleaved PARP (1:1000, ab32064, Abcam), cyclin D1 (1:150, 55506, Cell Signaling Technology), peEF2 (1:100, 2331, Cell Signaling Technology), p4E-BP1 Thr37/46 (1:250, 2855, Cell Signaling Technology), pS6(Ser235/236) (1:75, 4858, Cell Signaling Technology), ribosomal protein pS6(Ser240/244) (1:1000, 5364, Cell Signaling Technology), SOX9 (1:500, AB5535, Millipore) cMYC (1:800, ab32072, Abcam)

Validation

All antibodys were selected on the manufacturers recommendations regarding target species. All antibodies were optimised and validated on control tissue with known expression of target antigen; location and intensity of signal where assessed to confirm correct antibody binding.

Animals and other research organisms

Policy information about studies involving animals; ARRIVE guidelines recommended for reporting animal research, and Sex and Gender in Research

Laboratory animals

Mus Musculus with a mixed C57BL/6 background were used, experiments were started on mice aged 2–4 months.

Wild animals

Study did not involve wild animals

Reporting on sex

Mouse sex was determined at weaning by inspecting the genitalia. Experiments using the Gls2CreER only included males as there were differences in in genetic recombination between males and females. Experiments using 6.4×108 GC/ml only used males as the AAV8.TBG.Cre tropism is different between sexes. An exception to this was in the treatment of BrafV600E/+;Rnf43fl/fl;Znrf3fl/fl mice with dabrafenib and LGK974; here, equal numbers of males and females were used per experimental group. In all other animal experiments equal numbers of males and females were used in each experimental group.

Field-collected samples

Study did not involve samples collected from the field

Ethics oversight

All mouse experiments were performed according to UK Home Office regulations (project licence 70/8646 and PP3908577) following approval by the University of Glasgow Animal Welfare and Ethical Review Body.

Note that full information on the approval of the study protocol must also be provided in the manuscript.

Plants

Seed stocks

Report on the source of all seed stocks or other plant material used. If applicable, state the seed stock centre and catalogue number. If plant specimens were collected from the field, describe the collection location, date and sampling procedures.

Novel plant genotypes

Describe the methods by which all novel plant genotypes were produced. This includes those generated by transgenic approaches, gene editing, chemical/radiation-based mutagenesis and hybridization. For transgenic lines, describe the transformation method, the number of independent lines analyzed and the generation upon which experiments were performed. For gene-edited lines, describe the editor used, the endogenous sequence targeted for editing, the targeting guide RNA sequence (if applicable) and how the editor was applied.

Authentication

Describe any authentication procedures for each seed stock used or novel genotype generated. Describe any experiments used to assess the effect of a mutation and, where applicable, how potential secondary effects (e.g. second site T-DNA insertions, mosiacism, off-target gene editing) were examined.
